# Research on Person Re-Identification through Local and Global Attention Mechanisms and Combination Poolings

**DOI:** 10.3390/s24175638

**Published:** 2024-08-30

**Authors:** Jieqian Zhou, Shuai Zhao, Shengjie Li, Bo Cheng, Junliang Chen

**Affiliations:** State Key Laboratory of Networking and Switching Technology, Beijing University of Posts and Telecommunications, Beijing 100876, China; zhaoshuaiby@bupt.edu.cn (S.Z.); lishengjie@bupt.edu.cn (S.L.); chengbo@bupt.edu.cn (B.C.); chjl@bupt.edu.cn (J.C.)

**Keywords:** person re-identification, attention mechanism, combination pooling, max pooling, average pooling

## Abstract

This research proposes constructing a network used for person re-identification called MGNACP (Multiple Granularity Network with Attention Mechanisms and Combination Poolings). Based on the MGN (Multiple Granularity Network) that combines global and local features and the characteristics of the MGN branch, the MGNA (Multiple Granularity Network with Attentions) is designed by adding a channel attention mechanism to each global and local branch of the MGN. The MGNA, with attention mechanisms, learns the most identifiable information about global and local features to improve the person re-identification accuracy. Based on the constructed MGNA, a single pooling used in each branch is replaced by combination pooling to form MGNACP. The combination pooling parameters are the proportions of max pooling and average pooling in combination pooling. Through experiments, suitable combination pooling parameters are found, the advantages of max pooling and average pooling are preserved and enhanced, and the disadvantages of both types of pooling are overcome, so that poolings can achieve optimal results in MGNACP and improve the person re-identification accuracy. In experiments on the Market-1501 dataset, MGNACP achieved competitive experimental results; the values of mAP and top-1 are 88.82% and 95.46%. The experimental results demonstrate that MGNACP is a competitive person re-identification network, and that the attention mechanisms and combination poolings can significantly improve the person re-identification accuracy.

## 1. Introduction

The task of person re-identification aims to find images of the same person’s identity, in gallery of images, matched with query images [1]. A query image and an image matched of a person with the same identity have differences such as lighting, pose, viewpoint, occlusion, background clutter, etc., which makes person re-identification challenging [2,3,4,5].

Deep neural networks are often implemented for feature extraction in person re-identification. Some networks usually only focus on the global features of persons’ images for re-identification and ignore the local details. On the other hand, others just employ the local features of images and ignore the global features. The MGN [6] is a network used to conduct re-identifications with good identification accuracy, which combines global and local features and selects ResNet-50 as the backbone network to extract the basic features of an image, and then uses three branches to learn both the coarse-grained global features and the medium-grained and fine-grained local features. The combination of global and local features for person re-identification improves the person re-identification accuracy. The MGN with good identification accuracy has been chosen for research to further develop an advanced approach.

The attention mechanism helps to concentrate on important information in the data [7,8], and it is applied in various aspects [7,8]. When image recognition and person re-identification are under consideration, the attention mechanism focuses on important features in the images [9]. The enhancement of important features makes it easier to distinguish, match, and identify person images, so the accuracy of the image recognition and person re-identification can be improved [9].

The attention mechanism is often implemented to learn the global features of an image and find the most important and most recognizable feature regions of an entire image in image recognition and person re-identification. This article has conducted research, analysis, and experiments on the MGN and attention mechanism. The global and local features extracted by the MGN are the features of fixed areas in an image. The application of the attention mechanism in the global area is conducive to learning the most important features of an entire image. The application of the attention mechanism in the local area helps master the most important features in the local area of an image. The application of both the global and local features derived by the attention mechanisms together can further improve the accuracy of person re-identification.

Pooling is a common operation in convolutional neural networks (CNNs) [10]. Also, pooling plays an important role in the MGN used for person re-identification since the local features of the MGN branch are obtained by equally dividing the global features into upper, lower, and upper middle and lower parts by the pooling method. The features before the poolings are run are global, and the height H, width W, and dimension (number of channels) C of the features are not 1, and the features are represented by H × W × C. After the poolings are run, there exist global features and local features, and the width and height of all features are 1, the dimension is not 1, and the features are represented by 1 × 1 × C. If focused on both local and global features, only the dimensional (channel) attention mechanism can work.

The commonly used pooling methods include max pooling and average pooling, which have their advantages and disadvantages. The poolings used in the MGN are Max poolings. If max pooling and average pooling can be used together, and the appropriate combination proportions of two pooling methods can be found through experiments, the combination pooling can preserve the advantages of the two pooling methods and overcome the disadvantages of the two pooling methods; thus, it can improve the accuracy of person re-identification.

The network that uses the attention mechanisms to focus on global and local features of the branch is called the MGNA when the initial network MGN is chosen. Then, the network that uses combination poolings instead of max poolings is called MGNACP when the MGNA is chosen.

The research contributions can be summarized as follows:

A person re-identification network, the MGN, has been chosen and it integrates global and local features to achieve good accuracy in person re-identification.

By utilizing the attention mechanisms on both the global and local features of the MGN, the MGNA enhances the identifiable quality of features to improve person re-identification accuracy.

Combination poolings are used by MGNACP on the basis of the MGNA in place of max poolings in order to retain the advantages and overcome the disadvantages of max and average pooling, which can enhance person re-identification accuracy.

The remaining part of this article is structured as follows. Section 2 reviews the related works regarding feature representation, the attention mechanism, and pooling aspects. The methods are thoroughly explained in Section 3, which also includes the structure of the MGN network, the attention mechanism and MGNA network, max pooling, average pooling, combination pooling, and the MGNACP network. Section 4 compares the results of the proposed method with those of state-of-the-art approaches. The experimental results of the MGNA (MGN with attentions) were discussed. The parameter proportions of combination poolings were explored, and the best combination pooling parameters of MGNACP were found through experiments. The research is concluded in Section 5.

## 2. Related Work

### 2.1. Feature Representation

When person re-identification is under investigation, feature representation is a very important aspect. The better the learned feature representation, the higher the accuracy of person re-identification. The feature representation of person re-identification is divided into global features, local features, and both global and local features. In the global feature representation, ref. [11] utilized ResNet-50 as the backbone network and only used global features to perform re-identification through various available training techniques. Ref. [12] took pointwise convolution, depth convolution, and multi-feature flow to extract the features of different scales and aggregate these features for person re-identification. Ref. [13] constructed two peer-to-peer deep networks and trained them using mutual learning strategies for person identification. Ref. [14] employed Singular Vector Decomposition (SVD) to optimize deep representation learning and generate more discriminative vectors for person re-identification. Ref. [15] added a relationship-aware global attention mechanism to ResNet to enable the network to better extract global features and increase accuracy.

When local features are under consideration, ref. [16] divided the image into horizontally overlapped strips, and extracted features from these strips for person re-identification by implementing histogram, color extraction, and texture operators. Ref. [17] learned the individual local feature projection of each image sample according to the current data distribution, and projected all the samples into a common discriminant space for the similarity metric. Ref. [18] proposed a bilinear convolutional network to learn the multi-region features of images for person re-identification. Ref. [19] divided the image into two non-overlapping upper and lower parts, and used two convolutional networks to train classification on the upper and lower parts. During testing, the features of the upper and lower parts were combined to conduct the distance metric. In [20], the image was fixed and evenly divided into multiple non-overlapping parts. The features extracted from each part of the image were matched with the corresponding area for the metric. Outliers were assigned to the area with the most similar match.

Among global and local representations, ref. [21] adaptively learned discriminative global and local representations. Ref. [22] generated middle modal images from the images of different modalities by utilizing the middle modal generator, extracted the combined features of the original image and the middle modal image by dual-stream network, and then extracted their global and local features for person re-identification. Ref. [23] used partial features and a pyramid spatial pooling module to learn the identity and attributes of the person and perform person re-identification. In the data enhancement stage, ref. [24] converted low-resolution images into super-resolution images, and then the features were extracted from low-resolution and super-resolution images to obtain fusion features. Next, pose estimation was selected as a guide to extract global and local features for person re-identification. Ref. [25] built two branches after the backbone network, one for extracting global features, and the other for deriving global and local features for person re-identification. After extracting features from the backbone network, ref. [26] constructed two branches. One was used to extract global features, the other evenly split features into multiple parts. The part prediction alignment method is used in the local branch to align the predicted distributions between each part and then combine the global and local features for person re-identification. Ref. [27] constructs two branches. One branch extracted the global features and used the graph convolutional network to learn the image structure. The other branch extracted local features, utilized graph convolution to learn the spatial relationships of local features, and combined global and local features to person re-identification. Ref. [28] learned global features and local features, as well as the relationship between global and local features, to enhance the connections between local and global contexts. Ref. [29] used a horizontal pyramid to learn global and multi-scale local features, and then took max pooling and average pooling to generate each partial feature representation and concatenate them for person re-identification.

The MGN person re-identification network is chosen, which combines local and global features to identify person images and achieve good person re-identification results [6].

### 2.2. Attention Mechanism

The attention mechanism is a mechanism that focuses on important information in things and can be applied to many fields such as text mining [30,31], speech recognition [32], and image recognition [33,34]. For example, in computer vision, the attention mechanism also has many applications, including image classification [35], object detection [36], face recognition [37], 3D vision [38], and person re-identification [39,40]. There are some commonly used and representative attention mechanisms. In [34], the Recurrent Attention Model (RAM) uses Recurrent Neural Network (RNN) to generate attention mechanisms. In [33], the Spatial Transformer Network (STN) is a spatial attention mechanism, which learns invariance to translation, scaling, rotation, and other more generic warping methods to accurately learn various changes of input data. It also spatially transforms and aligns the input data adaptively, so that the more important areas of the image in space are paid attention to. Ref. [35] took Global Average Pooling (GAP) instead of a fully connected layer for classification when classifying images, which can minimize the number of parameters while maintaining classification accuracy. The scores generated by GAP functioned as weights to linearly assign a weight to each image in the feature channel for linear addition to obtain the Class Activation Map (CAM). The CAM generated by GAP was input into the original image to make the classification interpretable, that is, it can be observed which areas of an image are most discriminative. Ref. [41] introduced the squeeze and excitation network (SE), a channel attention mechanism, that uses global average pooling and a fully connected network to learn the correlation between global information and channels, and it focuses on important channels. Ref. [42] proposed the Dual Attention Network (DANet), which uses the self-attention mechanism to form two types of attention mechanisms: one is spatial attention and the other is channel one. The outputs of spatial attention and channel attention are summed to form a hybrid attention mechanism that focuses on both space and channels.

Specifically, when person re-identification operation is under consideration, ref. [43] proposed a Siamese learning architecture that is able to understand the identity-aware invariant representation of cross-view matching and simultaneously learn the attention mechanism and the attention consistency mechanism to identify the consistent attention regions in images of the same identity. Ref. [40] established a high-order attention module to learn more subtle information, enhance image discrimination, and enrich the image information. Ref. [44] fused semantic features at different levels to guide the generation of attention modules for person re-identification. Ref. [45] constructed multiple branches to learn the Class Activation Map (CAM) in different regions and proposed a ranking activation map, which helped obtain more discriminative features for person re-identification. Ref. [46] employed the Class Activation Map (CAM) as supervision information to generate spatial attention mechanisms, used the similarity between spatial attention mechanisms and channel feature maps as channel attention, and performed spatial and channel attention on the features extracted by the backbone network for person re-identification. Ref. [47] built a Siamese network to extract features from image pairs. After the backbone networks extracted features, they used the self-attention mechanism to focus on the important features of a single image and then applied the mutual attention mechanism to focus on the common features of image pairs for person re-identification. Ref. [48] combined an attention mechanism through a spatial attention module and channel attention module and applied it in the multi-layer convolutional network for person re-identification.

The implemented attention mechanisms are constructed based on the characteristics of the MGN, and they employ channel attention mechanisms that not only focus on important global information, but also on significant local information in each part. Different from the SE channel attention [41], the implemented attention mechanisms do not utilize the fully connected network but the convolutional network with H × W × C of 1 × 1 × C.

### 2.3. Pooling

Pooling is an important component of CNNs and can increase the image receptive field and allow the network to be better optimized while preventing overfitting and keeping the feature map from being deformed. The commonly used pooling methods include max pooling and average pooling. Different CNNs employ different pooling methods. LeNet [49] used average pooling, AlexNet [50] and VGG [51] used max pooling, and ref. [10] applied global average pooling in CNNs. GoogLeNet [52] implemented max pooling and global average pooling. DenseNet [53] utilized max pooling, average pooling, and global average pooling. ResNet [54] conducted max pooling and global average pooling.

In person re-identification, ref. [55] proposed a focused attention network with a lightweight and multi-scale scheme to learn multi-scale feature representation for person re-identification. When the data are input in, they are first augmented, and after the initial feature extraction is run by a convolutional network, the average pooling is employed and then sent to the multi-scale focused network. When person re-identification is performed, the features first perform global average pooling. Ref. [56] put forward a two-stream network, one branch worked for extracting features from the original RGB image data, and the other one was responsible for extracting features from color images generated by randomly exchanging channels, to reduce color interference and perform person re-identification. After the image features were extracted by the backbone network, global average pooling and global max pooling were utilized to sum the features, and then the features were extracted for person re-identification. Ref. [57] selected discriminative video fragments in the video through the correlation insight model, and then sent the video fragments to the discrepancy description network to generate the discrepancy descriptor for person re-identification in videos. When extracting features in a correlation insight model, max pooling was applied. Ref. [58] extracted global features by a multi-scale global attention module. Additionally, local feature extraction considers the continuity of each part, which makes global features more reasonable and local features more detailed. During feature extraction, multiple modules employed global average pooling and global max pooling to extract the features. In [59], there were three branch subnetworks after the backbone network was constructed. They included an accessory attribute network to extract personal decoration information, a body attribute network to describe the regional structure of a person, and a color attribute network to describe color features. A tree feature selection algorithm was applied to construct global features for person re-identification. In the accessory attribute network, global average pooling was performed on each local feature when extracting local features. The color attribute network also utilized global average pooling when extracting color features. In [60], the proposed network could divide global features into local features according to the posture module, and it could find the correlation between different sample postures through the self-attention and external attention modules. At the same time, it can also not be disturbed by posture samples with large variations. The interaction between self-attention and external attention modules and the pose-guided module relied on average pooling.

The pooling method in this article is used to implement combination pooling, which is not the simple addition of max pooling and average pooling, but identifies the most suitable proportions of two pooling methods through experiments and combines them. Thus, the two pooling operations complement each other to overcome disadvantages and preserve advantages.

## 3. Methods

This section introduces the MGN structure, attention mechanism method of the MGNA, and combination pooling method of MGNACP. Table 1 lists all the symbols used in this section.

### 3.1. MGN Network

**Figure 1:**
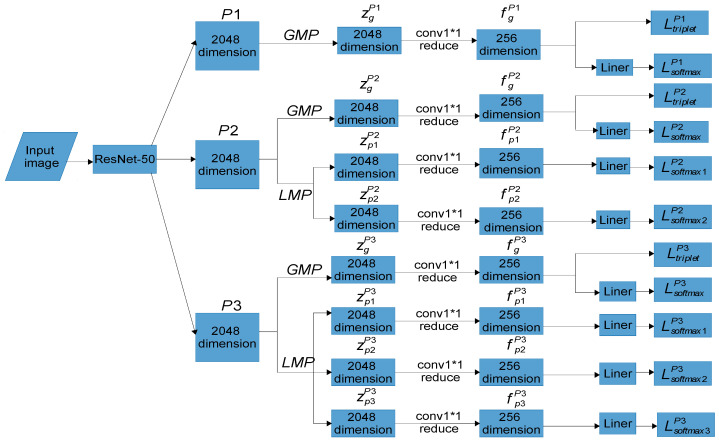
The overall architecture of the MGN. GMP: Global Max Pooling; LMP: Local Max Pooling; conv1*1 reduce: the features are reduced to 256 dimensions by a 1 × 1 convolution, and then subjected to batch normalization and ReLU activation function.

The Multiple Granularity Network (MGN) is a method for person re-identification combining multiple features and global features in images. Coarse-grained information is obtained from the global features of the image, while the medium-grained and fine-grained information are captured from multiple partial features of images. The MGN is composed of a backbone network and three branch networks. The backbone network is responsible for extracting the basic features of images through the ResNet-50 network with high recognition accuracy. The three branches further extract deeper semantic features of different granularities based on the features extracted by ResNet-50. The parameters of these three branches are not shared, but they share the ResNet-50 backbone network. The upper branch of the MGN extracts coarse-grained semantic information. The upper branch uses the Global Max Pooling (GMP), and the output features are then conducted with a 1 × 1 convolution, batch normalization, and ReLU activation functions. The final output dimension is 256. The middle branch of the MGN extracts medium-grained semantic information. The middle branch uses GMP and Local Max Pooling (LMP). The former is used to learn global features, and the latter evenly horizontally divides the feature into upper and lower stripes. Each output feature learns medium-grained semantic features in the same way as the upper branch setting. The lower branch extracts fine-grained semantic information and utilizes GMP and LMP. The former is not divided to learn global features, and the latter evenly and horizontally divides the feature into three stripes: upper, middle, and lower. Each output feature learns fine-grained semantic features in the same way as the middle-branch setting. During the testing process, the upper branch is equipped with a single 256-dimensional global feature, while the middle branch incorporates one 256-dimensional global feature and two 256-dimensional local features. Similarly, the lower branch includes one 256-dimensional global feature and three 256-dimensional local features. These concatenated global and local features serve as identification features for person re-identification. The three branches of the MGN are recorded as Pi, i=1, 2, 3. g denotes the global feature branch that passes through GMP, pj represents the local feature branch that passes through LMP, i=2, j=1, 2; i=3, j=1, 2, 3. The global and local features of the three branches after GMPs and LMPs are run are shown as $z_{g}^{\mathrm{Pi}}$, $z_{\mathrm{pj}}^{\mathrm{Pi}}$. It continues feature extraction to obtain 256-dimensional global and local features, which are recorded as $f_{g}^{\mathrm{Pi}}$, $f_{\mathrm{pj}}^{\mathrm{Pi}}$. In testing, $f_{g}^{\mathrm{Pi}}$ and $f_{\mathrm{pj}}^{\mathrm{Pi}}$ are concatenated for the distance metric for person re-identification. For more information on the MGN network, the MGN can be referred to [6]. The overall architecture of the MGN is demonstrated in Figure 1.

### 3.2. Attention Mechanism Method of MGNA

The three branches of the MGN after ResNet-50 form the three global features $z_{g}^{\mathrm{Pi}}$, i=1, 2, 3 and five local features $z_{\mathrm{pj}}^{\mathrm{Pi}}$, i=2, j=1, 2; i=3, j=1, 2, 3. Global features learn the overall information of the picture, and local features grasp the local information of the upper, lower, and upper middle and lower parts of the picture. The attention mechanisms learn the most discriminative information in the area of interest and focuses on this information. Although the MGN learns global and local features, global features are dedicated to learning global information, and local features are dedicated to learning local information. But there is also the most discriminating information in global and local information respectively. The attention mechanisms can learn the most discriminating features of global and local features and improve identification accuracy. The MGN has both global and local features only after GMPs and LMPs, so the attention mechanisms for both global and local features can only be added after GMPs and LMPs. The features after GMPs and LMPs are run are 1 × 1 × 2048 convolution, where 1 × 1 denotes the height and width of the feature, and 2048 represents the number of channels of the feature, so we only need to employ the channel attention mechanisms to focus the features.

The channel attention mechanism performs a 1 × 1 convolution (the feature size becomes 1 × 1 × C/r) on the input feature (the feature size is 1 × 1 × C), and the channel dimensions are reduced from 2048 to 128. After passing through the ReLU activation function (the feature size is 1 × 1 × C/r), another 1 × 1 convolution is performed (the feature size becomes 1 × 1 × C), and the channel dimensions are increased from 128 to 2048. Then, it passes through the Sigmoid activation function (the feature size is 1 × 1 × C), and the output value is the channel attention weight. The learned channel attention weights are multiplied with the input features by the channel-wise so that more attention is paid to the important feature information. C is the number of channels of the feature and r is the reduction ratio used to limit model complexity and aid the generalization of the attention mechanism. The global attention mechanism is defined by

(1) $$v_{g}^{\mathrm{Pi}}=\mathrm{Sigmoid}\left({\mathrm{conv}}_{1\times 1}\left(\mathrm{ReLU}\left({\mathrm{conv}}_{1\times 1}\left(z_{g}^{\mathrm{Pi}}\right)\right)\right)\right)\cdot z_{g}^{\mathrm{Pi}},\;i=1,\;2,\;3$$

The local attention mechanism is delineated by

(2) $$v_{\mathrm{pj}}^{\mathrm{Pi}}=\mathrm{Sigmoid}\left({\mathrm{conv}}_{1\times 1}\left(\mathrm{ReLU}\left({\mathrm{conv}}_{1\times 1}\left(z_{\mathrm{pj}}^{\mathrm{Pi}}\right)\right)\right)\right)\cdot z_{\mathrm{pj}}^{\mathrm{Pi}},\;i=2,\;j=1,\;2;\;i=3,\;j=1,\;2,\;3$$

**Figure 2:**
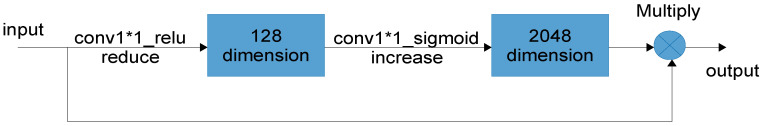
The schematic diagram of a channel attention mechanism. conv1*1_relu reduce: the feature is reduced to 128 dimensions by a 1 × 1 convolution, and then subjected to ReLU activation function; conv1*1_sigmoid increase: the feature is increased to 2048 dimensions by a 1 × 1 convolution, and then subjected to Sigmoid activation function.

The attention mechanism is shown in Figure 2.

The global features $v_{g}^{\mathrm{Pi}}$, i=1, 2, 3 and local features $v_{\mathrm{pj}}^{\mathrm{Pi}}$, i=2, j=1, 2; i=3, j=1, 2, 3 are obtained by the attention mechanisms, which continues to extract features. In the original MGN without the attention mechanism, the features are reduced from 2048 dimensions to 256 dimensions through performing a 1 × 1 convolution of the features $z_{g}^{\mathrm{Pi}}$ and $z_{\mathrm{pj}}^{\mathrm{Pi}}$, and then passing through batch normalization and ReLU activation function to obtain the features $f_{g}^{\mathrm{Pi}}$ and $f_{\mathrm{pj}}^{\mathrm{Pi}}$, which are delineated as

(3) $$f_{g}^{\mathrm{Pi}}{=\mathrm{conv}}_{1\times 1}\left(\mathrm{BN}\left(\mathrm{ReLU}\left(z_{g}^{\mathrm{Pi}}\right)\right)\right),\;i=1,2,3$$

(4) $$f_{\mathrm{pj}}^{\mathrm{Pi}}{=\mathrm{conv}}_{1\times 1}\left(\mathrm{BN}\left(\mathrm{ReLU}\left(z_{\mathrm{pj}}^{\mathrm{Pi}}\right)\right)\right),i=2,\;j=1,\;2;\;\;i=3,\;j=1,\;2,\;3$$

In the MGNA, the features are reduced from 2048 dimensions to 256 dimensions through performing a 1 × 1 convolution of the features $v_{g}^{\mathrm{Pi}}$ and $v_{\mathrm{pj}}^{\mathrm{Pi}}$, and then batch normalization and ReLU activation function are used to obtain features $f_{g}^{\mathrm{Pi}}$ and $f_{\mathrm{pj}}^{\mathrm{Pi}}$, which are delineated as

(5) $$f_{g}^{\mathrm{Pi}}{=\mathrm{conv}}_{1\times 1}\left(\mathrm{BN}\left(\mathrm{ReLU}\left(v_{g}^{\mathrm{Pi}}\right)\right)\right),\;i=1,\;2,\;3$$

(6) $$f_{\mathrm{pj}}^{\mathrm{Pi}}{=\mathrm{conv}}_{1\times 1}\left(\mathrm{BN}\left(\mathrm{ReLU}\left(v_{\mathrm{pj}}^{\mathrm{Pi}}\right)\right)\right),\;i=2,\;j=1,\;2;\;i=3,\;j=1,\;2,\;3$$

In testing, $f_{g}^{\mathrm{Pi}}$ and $f_{\mathrm{pj}}^{\mathrm{Pi}}$ are concatenated for the distance metric for person re-identification.

**Figure 3:**
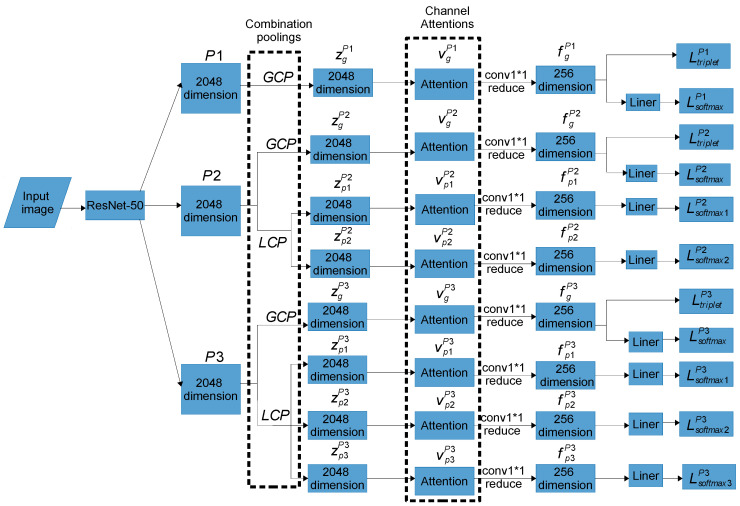
The overall architecture of MGNACP. GCP: Global Combination Pooling; LCP: Local Combination Pooling; Attention: a channel attention machine; conv1*1 reduce: the features are reduced to 256 dimensions by a 1 × 1 convolution, and then they are subjected to batch normalization and ReLU activation function; Combination Poolings: the combination pooling part of each branch of MGNACP, where GCP is used to obtain global features and LCP is used to obtain local features; Channel Attentions: the channel attention mechanism part of each branch of MGNACP, where each global and local branch adds a corresponding channel attention mechanism (the attention mechanism of each global branch is conducive to learning the most important information of the global feature. The attention mechanism of each local branch is conducive to learning the most important information of local feature).

The attention mechanisms are added to the MGN (including global and local branches) to learn the most discriminating information in global and local features. The method of adding attention mechanisms to the MGN branch is denoted by MGNA (Multiple Granularity Network with Attentions), as shown in Figure 3.

### 3.3. Combination Pooling Method of MGNACP 

The CNN (Convolution Neural Network) has a powerful feature learning ability and is widely implemented in various fields of artificial intelligence. Convolution and pooling are its two important components. The pooling operation has a fixed-size pooling window that can slide and calculate on the feature. When compared with convolution, after the parameters are fixed, the pooling operation does not learn the hyperparameters. The pooling operation is mainly employed in the spatial dimension to change the data size and reduce the size of the feature. The pooling operation retains substantial features and reduces redundant ones as well as the amount of calculation, so that the network can be better optimized while preventing overfitting. The pooling operation also increases the image receptive field. The size of the input feature area corresponding to one pixel of the output feature is the receptive field. When performing the pooling operation, the pooling window takes multiple pixels of the feature before pooling, which corresponds to one pixel after pooling, so the receptive field of the feature after pooling is increased. The pooling operation helps keep the feature invariant, including translation, scaling, and rotation invariant, which means that the input features with slight translation, scaling, and rotation are pooled, and the output results are the same. While max pooling takes the maximum value in the pooling area as output, average pooling computes the average value in the pooling area as output.

Pooling first determines the size of the pooling window and the stride size when the pooling window slides. The pooling value of each region that the pooling window slides through in the feature serves as the output feature value. $R_{f}$ represents the pooling area, the feature area where the pooling window is located. f indicates the sequence number of pooling areas which the feature is divided by the pooling window. F refers to the number of pooling areas which the feature is divided by the pooling window. $\left|R_{f}\right|$ denotes the number of pixels in the pooling area where the pooling window is located. i represents the ith pixel in the pooling area where the pooling window is located. $u_{i}$ describes the ith pixel value in the pooling area where the pooling window is located. R means the entire pooling area, that is, the feature area where the pooling window is located is the entire feature. $\left|R\right|$ indicates the number of pixels in the entire pooling area R. $v_{f}$ represents the output value after the pooling calculation of the fth pooling area $R_{f}$ in the feature. v designates the output value of the entire pooling area R after pooling calculation. $\lambda \in \left(0,\;1\right)$ denotes the proportion of max pooling and average pooling in combination pooling. λ is determined experimentally and discussed in Section 4.

(1) Max pooling

Max pooling is defined as:

(7) $$v_{f}=\underset{u_{i}\in R_{f}}{\max}u_{i},\;f=1,\;2,\;3,\cdots ,\;F,\;i=1,\;2,\;3,\cdots ,\left|R_{f}\right|$$

Max pooling can be divided into local max pooling and global max pooling. The pooling window is smaller than the size of the feature, and the feature is divided into several areas for max pooling, which is Local Max Pooling (LMP). The pooling window is the same size as the feature, the feature is not divided into several areas, and the entire feature is performed via max pooling, which is Global Max Pooling (GMP).

Global max pooling is defined as:

(8) $$v=\underset{\;u_{i}\in R}{\max\;}u_{i},\;i=1,\;2,\;3,\cdots ,\left|R\right|$$

(2) Average pooling

Average pooling is defined as:

(9) $$v_{f}=\frac{1}{\left|R_{f}\right|}\sum_{u_{i}\in R_{f}}u_{i},\;f=1,\;2,\;3,\cdots ,\;F,\;i=1,\;2,\;3,\cdots ,\left|R_{f}\right|$$

Average pooling can be divided into local average pooling and global average pooling. When the pooling window is smaller than the feature size, the feature is divided into several regions for average pooling, which is called Local Average Pooling (LAP). The pooling window is the same size as the feature, the feature is not divided into several regions, and the entire feature is performed via average pooling, which is Global Average Pooling (GAP).

Global average pooling is defined as:

(10) $$v=\frac{1}{\left|R\right|}\sum_{u_{i}\in R}u_{i},\;i=1,\;2,\;3,\cdots ,\left|R\right|$$

(3) Combination pooling

Max pooling selects the maximum pixel value of the pooling area as output, which can save the texture information of the image, but also ignores detailed information. Average pooling picks the average pixel value of the pooling area as the output, which can preserve the overall feature information, but also makes the features blurry and difficult to distinguish. By combining max pooling and average pooling, the most suitable proportions of two methods of pooling are determined through experiments, which not only preserve and enhance the advantages of two methods of pooling, but also overcome the shortcomings of two methods of pooling. Then, combination pooling is defined as:

(11) $$v_{f}=\lambda \cdot \frac{1}{\left|R_{f}\right|}\sum_{u_{i}\in R_{f}}u_{i}+\left(1-\lambda \right)\cdot \underset{\;u_{i}\in R_{f}}{\max}u_{i},\;f=1,\;2,\;3,\cdots ,\;F,\;i=1,\;2,\;3,\cdots ,\left|R_{f}\right|$$

Combination pooling can be divided into local combination pooling and global combination pooling. The pooling window is smaller than the size of the feature, and the feature is divided into several areas for combination pooling, which is Local Combination Pooling (LCP). The pooling window is the same size as the feature, the feature is not divided into several areas, and the entire feature is performed via combination pooling, which is Global Combination Pooling (GCP).

Combining global pooling is defined as:

(12) $$v=\lambda \cdot \frac{1}{\left|R\right|}\sum_{u_{i}\in R}u_{i}+\left(1-\lambda \right)\cdot \underset{\;u_{i}\in R}{\max\;}u_{i},\;i=1,\;2,\;3,\cdots ,\left|R\right|$$

**Figure 4:**
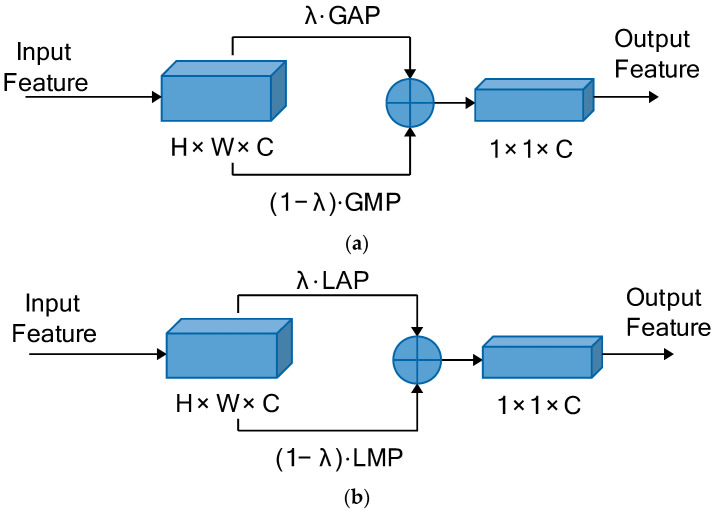
The schematic diagrams of GCP and LCP. (**a**) The schematic diagram of GCP. (**b**) The schematic diagram of LCP.

The combination poolings of the proposed method is derived based on the MGNA. The pooling part of each branch implements combination pooling instead of single pooling. Global Combination Pooling (GCP) is utilized in each branch of global pooling, and Local Combination Pooling (LCP) is implemented in each branch of local pooling. The parameters of combination pooling applied in each branch are the same. The proposed method is called MGNACP (Multiple Granularity Network with Attention Mechanisms and Combination Poolings). Through experiments, the most suitable parameters for combination poolings are determined, that is, the proportions of max pooling and average pooling in each combination pooling, which preserves and enhances the advantages of two pooling methods, overcomes the disadvantages, and makes the poolings achieve the best performance in the MGNACP. In experiments, we set the combination pooling parameter $\lambda \in \left(0,1\right)$, starting from λ=0.1 and incrementing to 0.9 in steps of 0.1. The optimal combination pooling parameters are the combination pooling parameters λ and 1−λ corresponding to the best experimental result of MGNACP. The schematic diagrams of single GCP and single LCP in MGNACP are shown in Figure 4. The overall architecture of MGNACP is shown in Figure 3.

## 4. Experimentation

### 4.1. Dataset and Evaluation Protocol

The experiment employs a large scale person re-identification dataset called the Market1501, which is collected by five 1280 × 1080 high-definition cameras and one 720 × 576 low-definition camera in different areas and different angles. The collection areas of some cameras have certain slight overlaps. Each identity is captured by at least two or more cameras. The dataset can use query images to find pictures of people with the same identity from different cameras. There are 12,936 training images, and the training set has 751 person identities in the Market1501 dataset. There are 19,732 test pictures, and the test set has 750 person identities. A total of 1501 person identities are employed for the experiment. There are 3368 query images in the dataset. The dataset has an average of 19.9 images per identity. The training and test sets of the dataset select the detection boxes detected by the person detector as annotation boxes, and the images in the query set utilize hand-drawn ground truth boxes as annotation boxes.

The experiment employs the CUHK03 dataset collected from two cameras. The dataset has two modes, one with hand-drawn labeled annotation boxes and one with detection boxes detected by the person detector as annotation boxes. The experiment selects the detection box data mode. There are 7365 training images, and the training set has 767 person identities. There are 5332 test images, and the test set has 700 person identities. A total of 1467 person identities are employed for the experiment. There are 1400 query images in the dataset. The dataset has an average of 9.7 images per identity.

The details of the datasets are shown in Table 2.

To compare the results of the leading methods with those of the proposed method, we use the mean Average Precision mAP and Cumulative Matching Characteristics (CMC) top-1 for comparison and evaluation.

### 4.2. Implementation Details

The experiment used an NVIDIA TITAN X GPU to accelerate the data process in parallel. The framework applied for the experiment is PyTorch. In the experiment, some parameters followed the settings of the original MGN [6], and the rest of the parameters have been improved from the original article. The input image size, data augmentation, and parameter initialization of ResNet-50 obey the original settings. The sampling parameters were improved in the training. Six identities were served for the training data sampled in each mini batch and four images were randomly selected for each identity. During training, the optimization was improved, that is, the ADAM optimizer was employed to train a total of 600 epochs. The initial learning rate is set to 0.0002. When training to 300 epochs, the learning rate decays to 0.00002. When training to 500 epochs, the learning rate decays to 0.000002.

### 4.3. Comparison with State-of-the-Art Methods

Table 3 reports the experimental results without re-ranking of person re-identification on the Market1501 dataset. We report from the perspective of the learning feature representation of person re-identification, including global features (represented by G), local features (represented by L), global and local multiple features (represented by M), and the learning feature method by attention mechanism (represented by A). In Table 3, the best results of all the experiments are listed in bold, and the gray background represents the best experimental results of each type of feature representation.

The experimental results of MGNACP are mAP of 88.82% and top-1 of 95.46%. For the experimental results of global feature representation, the method with the best mAP experimental results is the BoT, and the best method for the top-1 experimental results is the OSNet. In the experimental results of the BoT, mAP and top-1 are 85.9% and 94.5%. MGNACP is 2.92% higher than the BoT in mAP, and 0.96% higher than the BoT in top-1. In the experimental results of the OSNet, mAP is 84.9% and top-1 is 94.8%. MGNACP is 3.92% higher than OSNet in mAP, and 0.66% higher than the OSNet in top-1. For the experimental results of local feature representation, PCB + RPP presents the best experimental results, with a mAP value of 81.6% and a top-1 value of 93.8%. MGNACP is 7.22% higher than the PCB + RPP in mAP, and 1.66% higher than the PCB + RPP in top-1. For global and local multiple feature representations, the method with the best mAP experimental results is the PAGCN, and the best method for the top-1 experimental results is the MGN. In the experimental results of the PAGCN, mAP and top-1 are 87.3% and 94.4%. MGNACP is 1.52% higher than the PAGCN in mAP and 1.06% higher than the PAGCN in top-1. In the experimental results of the MGN, mAP is 86.9% and top-1 is 95.7%. MGNACP is 1.92% higher than the MGN in mAP, and 0.24% lower than the MGN in top-1. For methods that use attention mechanisms to learn feature representations, the method with the best mAP is AND, and the method with the best top-1 experimental results is MHN-6. In the experimental results of AND, mAP is 87.8% and top-1 is 92.3%. MGNACP is 1.02% higher than AND in mAP, and 3.16% higher than AND in top-1. In the experimental results of the MHN-6, mAP is 85.0% and top-1 is 95.1%. MGNACP is 3.82% higher than the MHN-6 in mAP, and 0.36% higher than the MHN-6 in top-1.

By comparing the proposed method with various methods used for person re-identification based on learned feature representations, MGNACP achieved a very good performance in the experimental results of the Market-1501 dataset. The experiment results of MGNACP are higher than the mAP of all the person re-identification methods shown in Table 3, and slightly lower than the top-1 experiment results of the MGN, but better than all of the other top-1 experiment results shown in Table 3. The experimental results indicate that the proposed method has achieved better person re-identification accuracy, showing that MGNACP is useful and feasible. In Section 4.4, the experimental results of the MGNA with the attention mechanisms and MGNACP with combination poolings are discussed more.

Table 4 reports the experimental results without re-ranking of person re-identification on the CUHK03 dataset using MGNACP and some competitive methods. In Table 4, the best experimental results are shown in bold. The mAP of MGNACP is 78.61%, and the top-1 is 81.57%. Among the experimental results, the better method is the MGN, whose mAP is 77.31% and the top-1 is 80.07%. MGNACP is 1.5% higher than the MGN in mAP and 1.3% higher than the MGN in top-1 results. Among the experimental results, MGNACP is better than all the experimental results, indicating that MGNACP is useful and feasible.

Table 5 reports the experimental results with re-ranking of person re-identification on the Market1501 dataset using MGNACP and some competitive methods. In Table 5, the best experimental results are shown in bold. Re-ranking has greatly improved the experimental results of all methods. The mAP of MGNACP with re-ranking is 94.55%, and the top-1 is 96.32%. Among the experimental results with re-ranking, the better method is the MGN, whose mAP is 94.2% and the top-1 is 96.6%. MGNACP is 0.35% higher than the MGN in mAP, and 0.28% lower than the MGN in top-1. Among the experimental results with re-ranking, MGNACP has the best mAP. Except that MGNACP is slightly lower than the MGN in top-1, it is better than all experimental results, indicating that MGNACP with re-ranking is useful and feasible.

Table 6 reports the experimental results with re-ranking of person re-identification on the CUHK03 dataset using MGNACP and some competitive methods. In Table 6, the best experimental results are shown in bold. Re-ranking has greatly improved the experimental results of all methods. The mAP of MGNACP with re-ranking is 87.82%, and the top-1 is 86.50%. Among the experimental results with re-ranking, the better method is the MGN, whose mAP is 87.02% and the top-1 is 86.07%. MGNACP is 0.8% higher than the MGN in mAP, and 0.43% higher than the MGN in top-1. Among the experimental results with re-ranking, the experimental results of MGNACP are better than all of the experimental results, indicating that MGNACP with re-ranking is useful and feasible. As can be seen from Table 4 and Table 6, for all methods in the CUHK03 dataset, in the experimental results without re-ranking, the top-1 experimental results are higher than the mAP experimental results; and in the experimental results with re-ranking, the mAP experimental results are higher than the top-1 experimental results.

**Figure 5:**
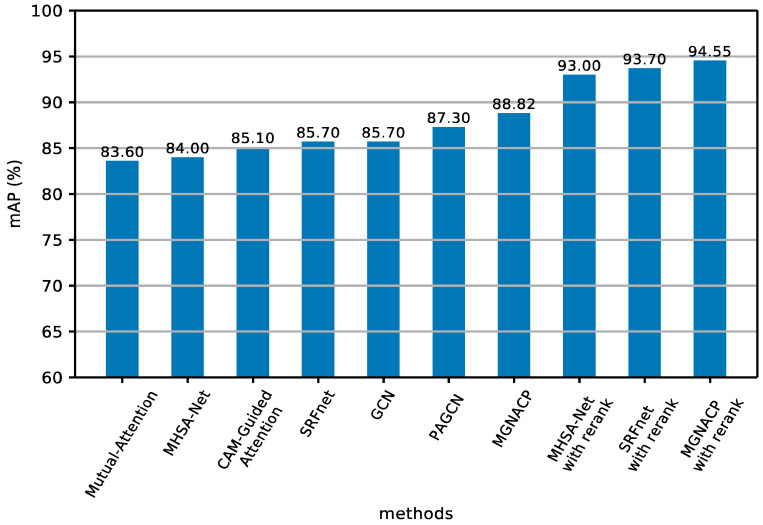
Histogram of results of mAP experiments on the Market-1501 dataset (including methods from recent years and MGNACP methods).

**Figure 6:**
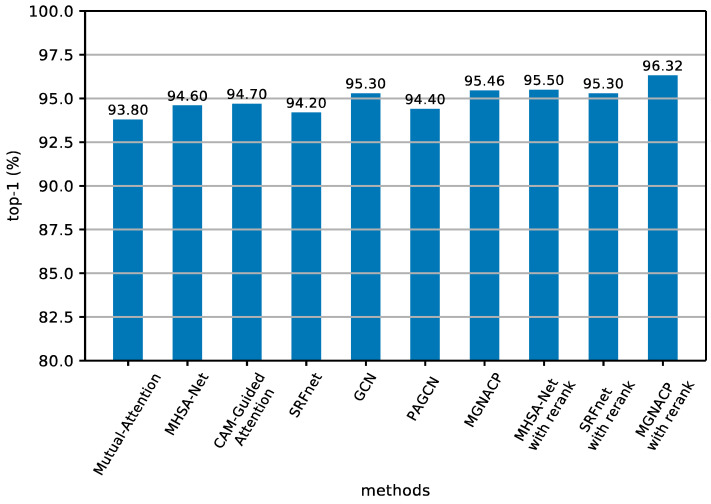
Histogram of results of top-1 experiments on the Market-1501 dataset (including methods from recent years and MGNACP methods).

Due to the fact that the experimental results of various methods of various types in the Market-1501 dataset are listed, it is not easy to view them directly. Therefore, Figure 5 and Figure 6 show the selection of the method with better experimental results in recent years from Table 3 and Table 5, as well as our method MGNACP. The results are displayed in histograms, so that the experimental results can be demonstrated more clearly. The mAP takes into account many factors, so it serves as the main evaluation basis for the experimental results. We arrange the mAP values of various methods from small to large. Figure 5 displays the mAP values, and Figure 6 draws the top-1 values, in which MGNACP has achieved the best mAP and top-1 scores with and without re-ranking.

**Figure 7:**
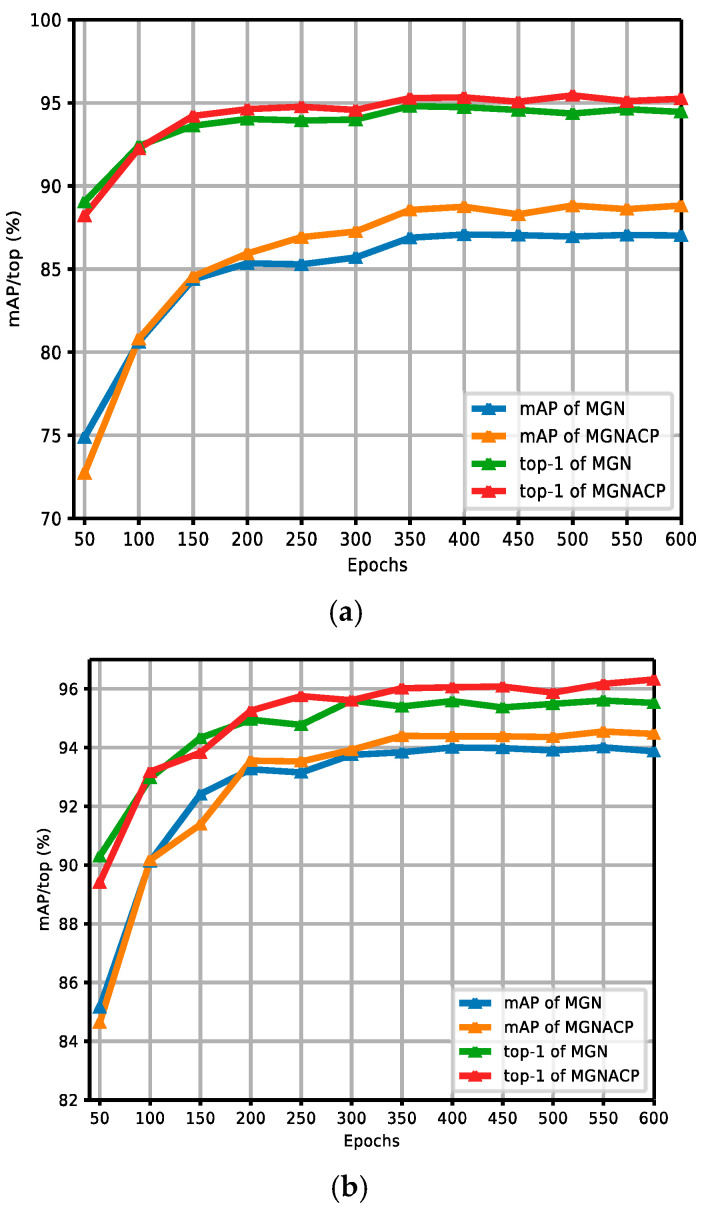
Comparison curves of experimental results between the MGN and MGNACP in the Market-1501 dataset without and with re-ranking. (**a**) Comparison curves of experimental results without re-ranking between the MGN and MGNACP. (**b**) Comparison curves of experimental results with re-ranking between the MGN and MGNACP.

Figure 7a,b show the comparison curves of experimental results between the MGN and MGNACP in the Market-1501 dataset without and with re-ranking. The figures report the experimental results of MGNACP in every 50 epochs of 600 training epochs, including mAP and top-1 values. As the training epoch continues to increase, the experimental results tend to increase steadily. When the experimental results increased steadily, MGNACP significantly outperformed the experimental results of the MGN in mAP and top-1, indicating that MGNACP (MGN with Attention Mechanisms and Combination Poolings) is useful and feasible. The gap between mAP and top-1 is narrowed through re-ranking.

**Figure 8:**
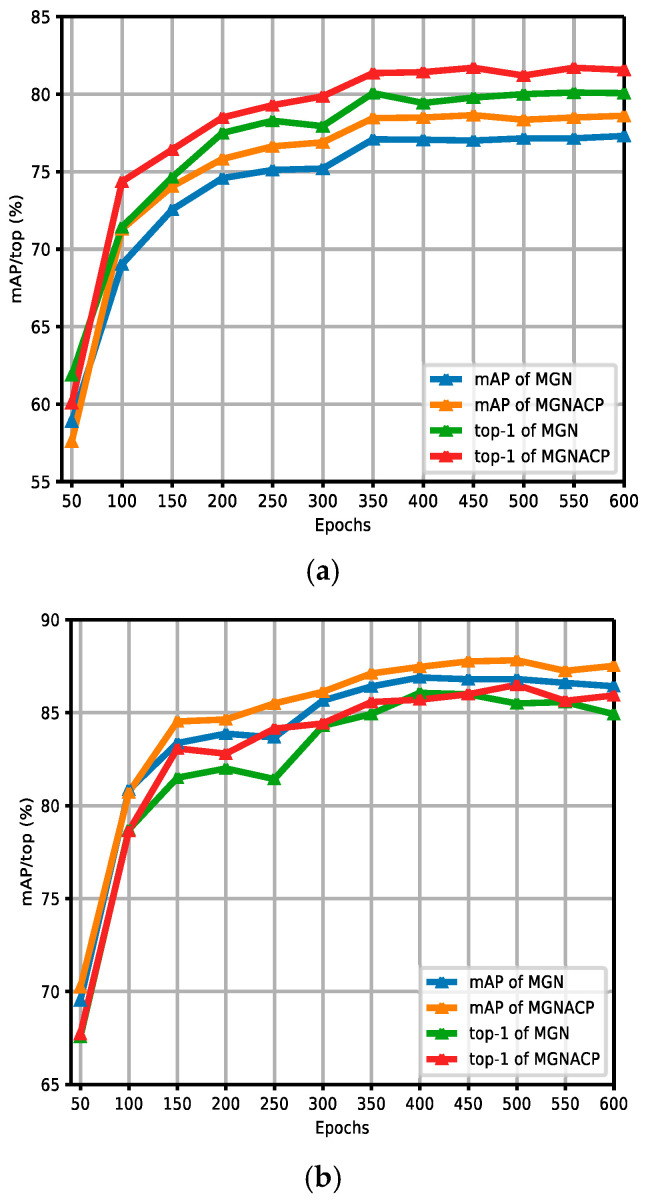
Comparison curves of experimental results between the MGN and MGNACP in the CUHK03 dataset without and with re-ranking. (**a**) Comparison curves of experimental results without re-ranking between the MGN and MGNACP. (**b**) Comparison curves of experimental results with re-ranking between the MGN and MGNACP.

Figure 8a,b show the comparison curves of the experimental results between the MGN and MGNACP in the CUHK03 dataset without and with re-ranking. The figures report the experimental results of MGNACP in every 50 epochs of 600 training epochs, including mAP and top-1 values. As the training epoch continues to increase, the experimental results tend to increase steadily. When the experimental results increased steadily, MGNACP significantly outperformed the experimental results of the MGN in mAP and top-1, indicating that MGNACP (MGN with Attention Mechanisms and Combination Poolings) is useful and feasible. The experimental results without re-ranking in Figure 8a show that there is a small gap between the results of mAP and top-1 experimental results. The re-ranking experimental results in Figure 8b show that when the experimental results tend to stabilize, mAP is higher than the top-1 experimental results.

### 4.4. Experimental Discussion

This section is the experimental discussion section, and we discuss the Attention Mechanisms and Combination Poolings of MGNACP.

#### 4.4.1. Experimental Results of Attention Mechanisms

In the attention mechanisms of MGNACP, we add attention mechanisms to each branch of the MGN (including global and local branches) to learn the most discriminative information in global and local features. The method of adding attention mechanisms to the MGN branches is called the MGNA. Then, we experiment with the attention mechanisms. In Table 7, the experimental results without re-ranking of the MGN and the MGNA on the Market-1501 dataset are listed. The mAP of the MGN is 86.9%, and the top-1 is 95.7%. The mAP of the MGNA is 88.46%, and the top-1 is 95.01%. The MGNA with the attention mechanisms is 1.56% higher than the MGN without the attention mechanism in mAP, and 0.69% lower in top-1.

Table 8 gives the experimental results with re-ranking of the MGN and the MGNA on the Market-1501 dataset. The mAP of the MGNA is 94.33%, and the top-1 is 95.93%. The mAP of the MGN is 94.2%, and the top-1 is 96.6%. The MGNA is 0.13% higher than the MGN in mAP, and 0.67% lower in top-1. From the experiment, it can be seen that the mAP improvement of the MGNA without re-ranking is greater, but the mAP of the MGNA with and without re-ranking has improved, which manifests that the proposed method suggesting the addition of the attention mechanisms to the MGN is useful and feasible.

**Figure 9:**
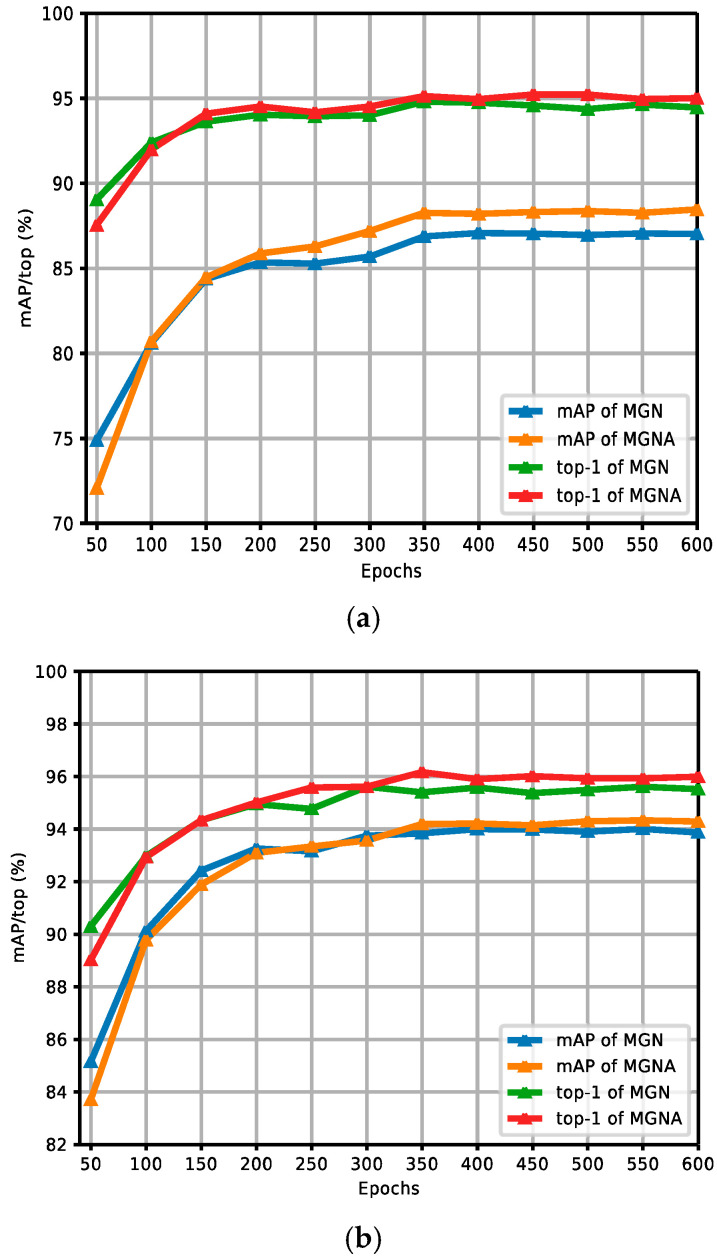
Comparison curves of experimental results between the MGN and MGNA in the Market-1501 dataset without and with re-ranking. (**a**) Comparison curves of experimental results without re-ranking between the MGN and MGNA. (**b**) Comparison curves of experimental results with re-ranking between the MGN and MGNA.

Figure 9a,b show the comparison curves of the experimental results between the MGN and MGNA in the Market-1501 dataset without and with re-ranking. The figures report the experimental results of MGNACP in every 50 epochs of 600 training epochs, including mAP and top-1 values. As the training epoch continues to increase, the experimental results tend to increase steadily. When the experimental results increased steadily, MGNACP outperformed the experimental results of the MGN in mAP and top-1, indicating that the MGNA (MGN with Attentions) is useful and feasible. The gap between mAP and top-1 is narrowed through re-ranking.

In Table 9, the experimental results without re-ranking of the MGN and the MGNA on the CUHK03 dataset are listed. The mAP of the MGN is 77.31%, and the top-1 is 80.07%. The mAP of the MGNA is 77.53%, and the top-1 is 80.79%. The MGNA with the attention mechanisms is 0.22% higher than the MGN without the attention mechanism in mAP, and 0.72% higher in top-1.

Table 10 gives the experimental results with re-ranking of the MGN and the MGNA on the CUHK03 dataset. The mAP of the MGNA is 87.38%, and the top-1 is 86.50%. The mAP of the MGN is 87.02%, and the top-1 is 86.07%. The MGNA is 0.36% higher than the MGN in mAP, and 0.43% higher in top-1. From the experiment, it can be seen that the mAP of the MGNA with and without re-ranking has improved, which manifests that the proposed method suggesting the addition of the attention mechanisms to the MGN is useful and feasible.

**Figure 10:**
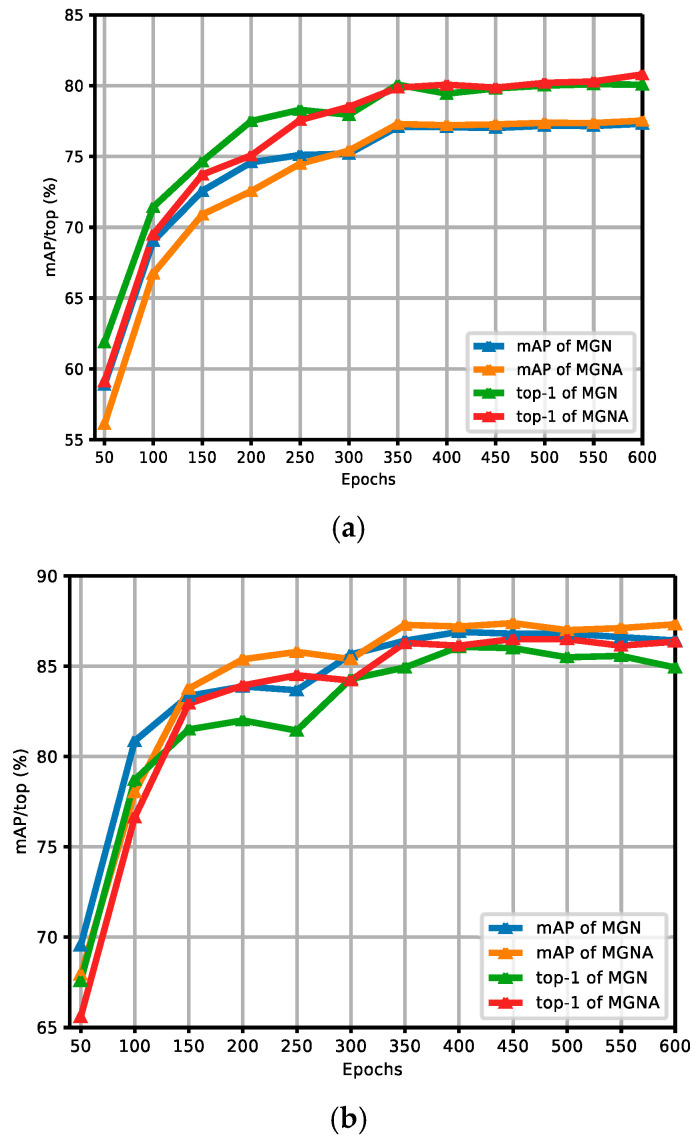
Comparison curves of experimental results between the MGN and MGNA in the CUHK03 dataset without and with re-ranking. (**a**) Comparison curves of experimental results without re-ranking between the MGN and MGNA. (**b**) Comparison curves of experimental results with re-ranking between the MGN and MGNA.

Figure 10a,b show the comparison curves of the experimental results between the MGN and MGNA in the CUHK03 dataset without and with re-ranking. The figures report the experimental results of MGNACP in every 50 epochs of 600 training epochs, including mAP and top-1 values. As the training epoch continues to increase, the experimental results tend to increase steadily. When the experimental results increased steadily, the MGNA outperformed the experimental results of the MGN in mAP and top-1, indicating that the MGNA (MGN with Attentions) is useful and feasible. The experimental results without re-ranking in Figure 10a show that there is a small gap between the results of mAP and top-1 experimental results. The re-ranking experimental results in Figure 10b show that when the experimental results tend to stabilize, mAP is higher than the top-1 experimental results.

#### 4.4.2. Experimental Results of Combination Poolings

For the combination poolings of MGNACP, the attention mechanisms are added to the MGN to form the MGNA and applied the combination pooling for each branch instead of using a single pooling for each branch. We found the most suitable parameters of combination poolings, that is, the proportions of the max pooling and the average pooling in each combination pooling are determined so that poolings can achieve the best performance in MGNACP through experimentation. In the experiments of each dataset, Max and Avg represent the proportions of max pooling and average pooling in combination pooling, with a change step size of 0.1 for both. There are nine groups of experiments in total, the combination pooling parameters corresponding to the best MGNACP experimental result are the optimal combination pooling parameters.

**Figure 11:**
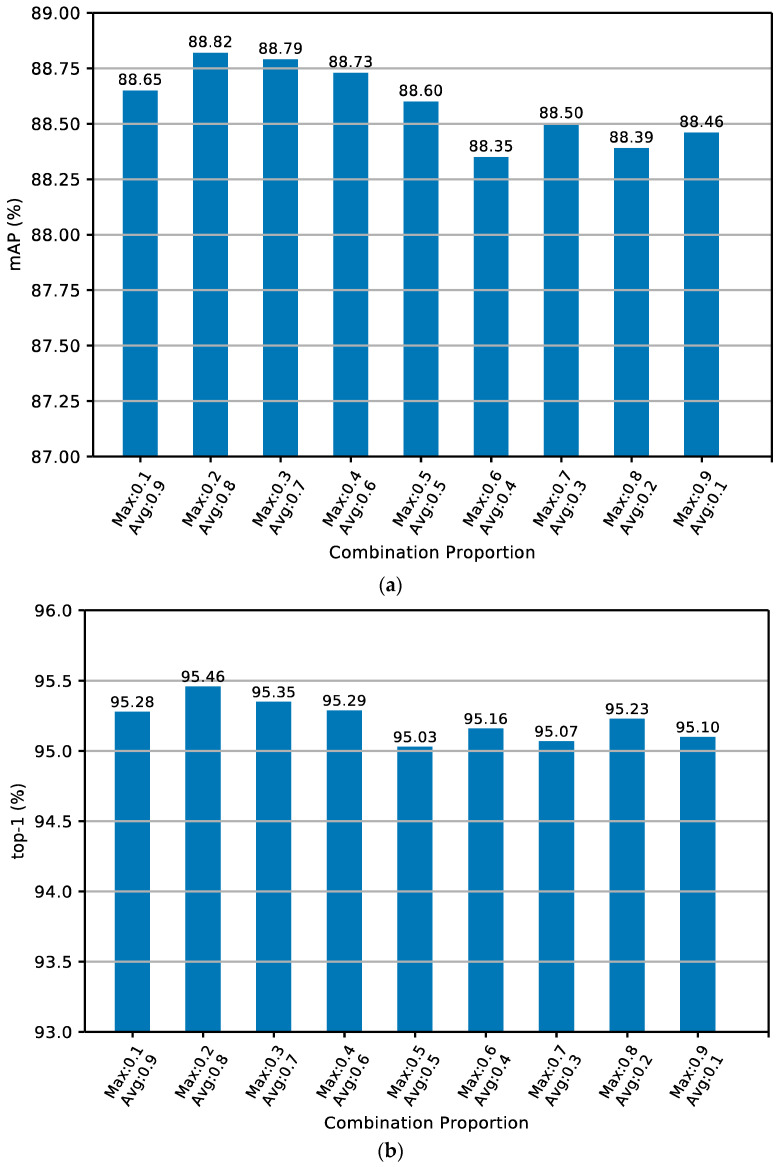
Histogram of the experimental results of top-1 and mAP for MGNACP on the Market-1501 dataset. (**a**) Histogram of the experimental results of mAP. (**b**) Histogram of the experimental results of top-1.

Table 11 depicts that the experimental results for the combination pooling parameters of MGNACP on the Market-1501 dataset are listed. The best results of the experiment are shown in bold. The best experimental results are realized when the max pooling and average pooling account for 0.2 and 0.8 in each combination pooling. Under optimal combination poolings, the experimental result of mAP is 88.82%, and the experimental result of top-1 is 95.46%. The lowest result of mAP under the combination poolings experiment is 88.35%, and the lowest top-1 is 95.03%. The optimal result is 0.47% higher than the lowest result in mAP, and 0.43% higher in top-1. Therefore, the optimal solution for each combination pooling on the Market-1501 dataset is a max pooling proportion of 0.2 and an average pooling proportion of 0.8. Figure 11a,b show the mAP and top-1 experimental results the histogram of MGNACP on the Market-1501 dataset with different combination pooling parameters. The combination pooling parameters of MGNACP on the Market-1501 dataset reach the best when the max pooling and the average pooling proportions are 0.2 and 0.8. The experimental results of mAP and top-1 become the best.

**Figure 12:**
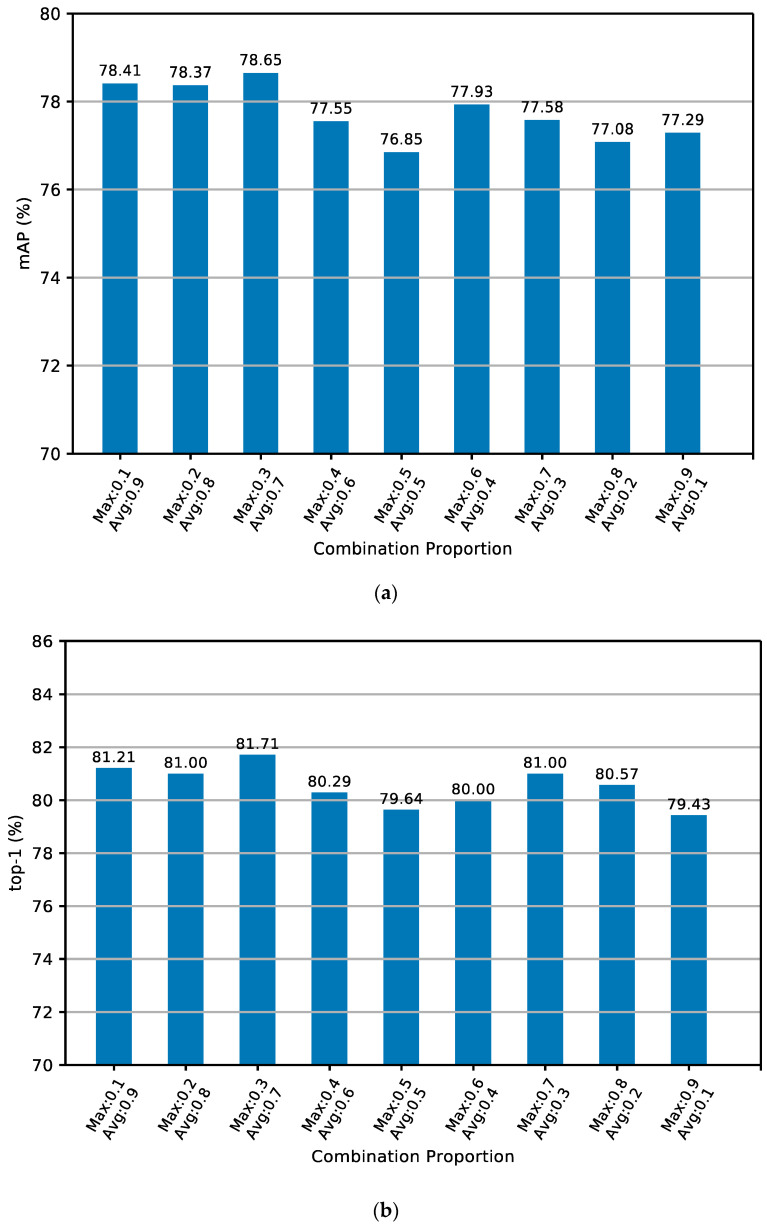
Histogram of the experimental results of top-1 and mAP for MGNACP on the CUHK03 dataset. (**a**) Histogram of the experimental results of mAP. (**b**) Histogram of the experimental results of top-1.

Table 12 depicts the experimental results for the combination pooling parameters of MGNACP on the CUHK03 dataset. The best results of the experiment are shown in bold. The best experimental results are realized when the max pooling and average pooling account for 0.3 and 0.7 in each combination pooling. Under optimal combination poolings, the experimental result of mAP is 78.65%, and the experimental result of top-1 is 81.71%. The lowest result of mAP under the combination poolings experiment is 76.85%, and the lowest top-1 is 79.43%. The optimal result is 1.8% higher than the lowest result in mAP, and 2.28% higher in top-1. Therefore, the optimal solution for each combination pooling on the CUHK03 dataset is a max pooling proportion of 0.3 and an average pooling proportion of 0.7. Figure 12a,b show the mAP and top-1 experimental results of the histogram of MGNACP on the CUHK03 dataset with different combination pooling parameters. The combination pooling parameters of MGNACP on the CUHK03 dataset reach the best when the max pooling and the average pooling proportions are 0.3 and 0.7. The experimental results of mAP and top-1 become the best.

From the two groups of experiments, it can be seen that the combined pooling parameters (Max, Avg) achieved good experimental results at (0.1, 0.9), (0.2, 0.8), and (0.3, 0.7).

## 5. Conclusions

This study constructs MGNACP used for person re-identification. To do so, an attention mechanism is added to each global and local branch of the MGN to construct the MGNA. Then, changing the single pooling of each branch to combination pooling forms MGNACP. MGNACP by attention mechanisms learns the most discriminative information of each global and local branch, and Combination Poolings preserve and enhance the advantages of max poolings and average poolings and overcome their shortcomings, so that the performance of poolings on MGNACP is optimized, and the accuracy of person re-identification is improved. MGNACP achieves high re-identification accuracy, indicating promising outcomes. In the future, we may develop a more advanced method for person re-identification by using more advanced attention mechanisms, different pooling methods, and distance metrics regarding the characteristics of images.

## Figures and Tables

**Table 1:** Symbols used in Section 3.

Symbols	Explanations
Pi, i=1, 2, 3	Branches of the MGN or branches of the MGNA or branches of the MGNACP
g	In the MGN and MGNA, it is the global feature branch using GMP or In MGNACP, it is the global feature branch using GCP
pj, j=1, 2 or j=1, 2, 3	In the MGN and MGNA, it is the local feature branch using LMP or In MGNACP, it is the local feature branch using LCP When branch i=2, the number of stripe j=1, 2; when branch i=3, the number of stripes j=1, 2, 3
$z_{g}^{\mathrm{Pi}}$, i=1, 2, 3	The features obtained by using GMP in each of the three branches of the MGN or The features obtained by using GCP in each of the three branches of MGNACP
$z_{\mathrm{pj}}^{\mathrm{Pi}}$, i=2, 3 then i=2, j=1, 2 then i=3, j=1, 2, 3	The features obtained by using LMPs in the P2 and P3 branches of MGNACP or The features obtained by using LCPs in the P2 and P3 branches of MGNACP
$f_{g}^{\mathrm{Pi}}$, i=1, 2, 3	In the MGN, global features $z_{g}^{\mathrm{Pi}}$ continue to extract features to obtain three 256-dimensional global features In MGNACP, global features $v_{g}^{\mathrm{Pi}}$ continue to extract features to obtain three 256-dimensional global features
$f_{\mathrm{pj}}^{\mathrm{Pi}}$, i=2, 3, then i=2*,*j=1, 2 then i=3, j=1, 2, 3	In the MGN, local features $z_{\mathrm{pj}}^{\mathrm{Pi}}$ further extract features to obtain five 256-dimensional local features In MGNACP, local features $v_{\mathrm{pj}}^{\mathrm{Pi}}$ further extract features to obtain five 256-dimensional global features
${\mathrm{conv}}_{1\times 1}$	1 × 1 convolution
BN	Batch normalization
ReLU	ReLU activation function
Sigmoid	Sigmoid activation function
$v_{g}^{\mathrm{Pi}}$, i=1, 2, 3	Global features obtained through the attention mechanisms
$v_{\mathrm{pj}}^{\mathrm{Pi}}$, i=2, 3 then i=2, j=1, 2 then i=3, j=1, 2, 3	Local features obtained through the attention mechanisms
$R_{f}$, f=1, 2, 3,⋯, F	Pooling area, the feature area where the pooling window is located
f, f=1, 2, 3,⋯, F	The sequence number of the pooling area that the feature is divided by the pooling window
F	The number of pooling areas that the feature is divided by the pooling window
$\left|R_{f}\right|$, f=1, 2, 3,⋯, F	The number of pixels in the pooling area where the pooling window is located
i, $i=1,\;2,\;3,\cdots ,\left|R\right|$	The ith pixel in the pooling area where the pooling window is located
$u_{i}$, $i=1,\;2,\;3,\cdots ,\left|R\right|$	The ith pixel value of the pooling area where the pooling window is located
R	The entire pooling area, that is, the feature area where the pooling window is located is the entire feature
$\left|R\right|$	The number of pixels in the entire pooling area R
$v_{f}$, f=1, 2, 3,⋯, F	The output value after the pooling calculation of the fth pooling area $R_{f}$ in the feature
v	The output value of the entire pooling area R after the pooling calculation
λ	$\lambda \in \left(0,\;1\right)$, the proportion of max pooling and average pooling in combination pooling
GMP	Global Max Pooling
LMP	Local Max Pooling
GAP	Global Average Pooling
LAP	Local Average Pooling
GCP	Global Combination Pooling
LCP	Local Combination Pooling
MGN	Multiple Granularity Network
MGNA	Multiple Granularity Network with Attentions
MGNACP	Multiple Granularity Network with Attention Mechanisms and Combination Poolings

**Table 2:** The details of datasets used in the experiments.

Detail	Market-1501	CUHK03
ID	1501	1467
Annotated box	32,668	14,096
Query box	3368	1400
Box per ID	19.9	9.7
Train box	12,936	7365
Test box	19,732	5332
Train ID	751	767
Test ID	750	700
Camera	6	2

**Table 3:** The comparison of experimental results between the state-of-the-art methods and the proposed method on the Market-1501 dataset. G: global features, L: local features, M: global and local multiple features, A: learning feature method by attention mechanism; the best results of all the experiments are listed in bold, and the gray background represents the best experimental results of each type of feature representation.

	Method	Top-1 (%)	mAP (%)
G	DML (2019) [13]	89.3	70.5
	OSNet (2019) [12]	94.8	84.9
	SVDNet (2017) [14]	82.3	62.1
	AOS (2018) [5]	86.5	70.4
	BoT (2019) [11]	94.5	85.9
L	PCB + RPP (2018) [20]	93.8	81.6
	PCB (2018) [20]	92.3	77.4
	Multi-region CNN (2017) [18]	41.2	66.4
	DLFOS + XQDA (2020) [16]	62.7	-
	part-based CNN + XQDA (2018) [19]	83.1	61.7
M	MSP-CNN (2019) [21]	84.2	66.3
	SR-DSFF + FENet-ReID (2022) [24]	90.9	-
	SRFnet (2023) [25]	94.2	85.7
	PPA + TS (2021) [26]	92.4	79.6
	PointReIDNet (2024) [61]	90.6	75.3
	PAGCN (2022) [27]	94.4	87.3
	GCN (2022) [28]	95.3	85.7
	HPM (2020) [29]	94.2	82.7
	PCN + PSP (2018) [23]	92.8	78.8
	MGN (2018) [6]	**95.7**	86.9
	DCR (2021) [4]	93.8	84.7
A	CASN (2018) [43]	94.4	82.8
	CAM-Guided Attention (2022) [46]	94.7	85.1
	Mutual-Attention (2020) [47]	93.8	83.6
	IANet (2019) [48]	94.4	83.1
	MHSA-Net (2022) [3]	94.6	84.0
	CLRA-CNN (2020) [44]	92.3	78.2
	AND (2022) [62]	92.3	87.8
	MHN-6 (2019) [40]	95.1	85.0
	PGFA (2019) [63]	91.2	76.8
	CAMA (2020) [45]	94.7	84.5
	HA-CNN (2018) [39]	91.2	75.7
	AL-APR (2021) [64]	89.0	74.4
	MGNACP (ours)	95.46	**88.82**

**Table 4:** The comparison of experimental results between the state-of-the-art methods and the proposed method on the CUHK03 dataset. The best experimental results are shown in bold.

Method	Top-1 (%)	mAP (%)
FMN (2020) [65]	42.6	39.2
PointReIDNet (2024) [61]	53.43	48.76
PCN + PSP (2018) [23]	60.7	56.0
AND (2022) [62]	60.6	56.5
HPM (2020) [29]	63.9	57.5
DCR (2021) [4]	68.4	61.4
PPA + TS (2021) [26]	65.5	62.4
CAMA (2020) [45]	66.6	64.2
CASN (2018) [43]	71.5	64.4
MHN-6 (2019) [40]	71.7	65.4
OSNet (2019) [12]	72.3	67.8
SRFnet (2023) [25]	73.3	69.6
MHSA-Net (2022) [3]	73.4	70.2
PAGCN (2022) [27]	75.1	71.6
GCN (2022) [28]	78.5	74.7
MGN(2018) [6] (Our Imp.)	80.07	77.31
MGNACP (ours)	**81.57**	**78.61**

**Table 5:** The comparison of experimental results with re-ranking between the state-of-the-art and the proposed method on the Market-1501 dataset. The best experimental results are shown in bold. CC* denotes the result with the official CC code without hard instance memory updating mechanism and generalized mean pooling [66].

Method	Top-1 (%)	mAP (%)
SRFnet (2023) [25]	95.3	93.7
PAGCN (2022) [27]	96.1	94.1
CAM-guided Attention (2022) [46]	95.1	92.7
MHSA-Net (2022) [3]	95.5	93.0
PCN + PSP (2018) [23]	94.4	90.8
MGN (2018) [6]	**96.6**	94.2
BoT (2019) [11]	95.4	94.2
SPReID (2018) [67]	94.6	91.0
FMN (2020) [65]	87.9	80.6
CC* + CAJ (2024) [66]	93.7	90.2
MV-3DSReID (2023) [38]	96.1	90.9
MGNACP (ours)	96.32	**94.55**

**Table 6:** The comparison of experimental results with re-ranking between the state-of-the-art and the proposed method on the CUHK03 dataset. The best experimental results are shown in bold.

Method	Top-1 (%)	mAP (%)
FMN (2020) [65]	47.5	48.5
PCN + PSP (2018) [23]	71.2	72.1
MHSA-Net (2022) [3]	80.2	80.9
SRFnet (2023) [25]	80.2	81.9
MGN (2018) [6] (Our Imp.)	86.07	87.02
MGNACP (ours)	**86.50**	**87.82**

**Table 7:** The experimental results without re-ranking of the MGN and the MGNA on the Market-1501 dataset.

Method	Top-1 (%)	mAP (%)
MGN	95.7	86.9
MGNA	95.01	88.46

**Table 8:** The experimental results with re-ranking of the MGN and the MGNA on the Market-1501 dataset.

Method	Top-1 (%)	mAP (%)
MGN	96.6	94.2
MGNA	95.93	94.33

**Table 9:** The experimental results without re-ranking of the MGN and the MGNA on the CUHK03 dataset.

Method	Top-1 (%)	mAP (%)
MGN	80.07	77.31
MGNA	80.79	77.53

**Table 10:** The experimental results with re-ranking of the MGN and the MGNA on the CUHK03 dataset.

Method	Top-1 (%)	mAP (%)
MGN	86.07	87.02
MGNA	86.50	87.38

**Table 11:** The experimental results of the parameter proportions of combination poolings on the Market-1501 dataset. The best experimental results are shown in bold.

Combination Proportion	Top-1 (%)	mAP (%)
Max: 0.1, Avg: 0.9	95.28	88.65
Max: 0.2, Avg: 0.8	**95.46**	**88.82**
Max: 0.3, Avg: 0.7	95.35	88.79
Max: 0.4, Avg: 0.6	95.29	88.73
Max: 0.5, Avg: 0.5	95.03	88.60
Max: 0.6, Avg: 0.4	95.16	88.35
Max: 0.7, Avg: 0.3	95.07	88.50
Max: 0.8, Avg: 0.2	95.23	88.39
Max: 0.9, Avg: 0.1	95.10	88.46

**Table 12:** The experimental results of the parameter proportions of combination poolings on the CUHK03 dataset. The best experimental results are shown in bold.

Combination Proportion	Top-1 (%)	mAP (%)
Max: 0.1, Avg: 0.9	81.21	78.41
Max: 0.2, Avg: 0.8	81.00	78.37
Max: 0.3, Avg: 0.7	**81.71**	**78.65**
Max: 0.4, Avg: 0.6	80.29	77.55
Max: 0.5, Avg: 0.5	79.64	76.85
Max: 0.6, Avg: 0.4	80.00	77.93
Max: 0.7, Avg: 0.3	81.00	77.58
Max: 0.8, Avg: 0.2	80.57	77.08
Max: 0.9, Avg: 0.1	79.43	77.29

## Data Availability

This article has no new dataset are created.
